# A Case Study of Aberrant Aortic Origin of the Right Coronary Artery

**DOI:** 10.15190/d.2024.3

**Published:** 2024-03-31

**Authors:** Jenab Hojefa Badodiyawala, Arpita Meher, Raju Shah, Bipin Chandra Aditya Dasari

**Affiliations:** ^1^Department of Medicine, David Tvildiani Medical University, Tbilisi, Georgia; ^2^Department of Medicine, Tbilisi State Medical University, Tbilisi Georgia; ^3^BP Koirala Institute of Health Science, Dharan, Nepal; ^4^Interventional Cardiologist, Apollo Hospitals Chennai Greams Road, India

**Keywords:** Aberrant Aortic Origin of the Right Coronary Artery (AAORCA), Right Coronary Artery Anomaly, Cardiovascular Anomaly, Coronary Angioplasty

## Abstract

This case report examines a rare cardiovascular abnormality, the Aberrant Aortic Origin of the Right Coronary Artery (AAORCA), in a 75-year-old patient with a history of myocardial infarction, acute renal injury, and cardiogenic shock. Rapid medical intervention, including coronary angioplasty, demonstrated the significance of prompt care. Chronic issues, including tobacco use and left ventricular dysfunction, complicated matters, emphasizing the importance of comprehensive long- term therapy. This study underscores the critical clinical significance of AAORCA (Anomalous aortic origin of the right coronary artery) following the SCARE 2023 reporting criteria. This abstract emphasizes the delicate relationship between congenital defects, chronic hazards, and proactive healthcare in complex cardiovascular situations.

## INTRODUCTION

It has been discovered that the incidence of aberrant aortic origin of the coronary arteries is lower, at 0.64 percent of births^1^. The prevalence of either the left main coronary artery originating from the right sinus of Valsalva or the RCA emerging from the left sinus of Valsalva is 0.1-0.3%^2^. In otherwise healthy people, variations in the amount, shape, and source of the coronary arteries, or ostia, can occur. It appears that the vast majority of these alterations have little clinical relevance^3^. Myocardial ischemia and sudden death can also occur when the RCA originates from the left sinus of Valsalva, generally known as the LAD^4^.

This case report focuses on a 75-year-old man with a complex medical history who presents with exertional chest discomfort, dyspnea, and diaphoresis in a private practice setting. Highlighting the clinical importance of AAORCA (Aberrant aortic origin of the right coronary artery).

## CASE PRESENTATION

A 75-year-old man presented at the emergency hospital after feeling exertional chest discomfort, dyspnea, and diaphoresis over the past 15-20 days. The chest discomfort was described as crushing, substernal, dull, and painful with heaviness in the chest, which was alleviated with nitrates radiating to the neck. His risk factor was tobacco usage during the past two decades.

The patient had a long medical history, comprising a non-ST elevation myocardial infarction, acute renal damage, and an episode of cardiogenic shock. In 2020, PTCA (percutaneous transluminal coronary angioplasty) was performed to revascularize the LAD.

Upon admission, the patient's vital values were 90/60 mmHg and 68 bpm. The chest was clean on both sides, and a lung test indicated no abnormalities. The heart was examined and found to be free of murmurs. The respiratory rate was 20 breaths per minute, and SPO2 was 98%. The patient was aware of his surroundings.

**Figure 1:**
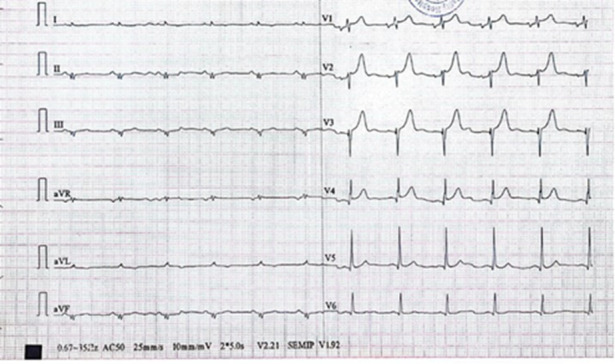
Figure 1. Initial presentation of the electrocardiogram with a heart rate of 68 and Hyperacute T wave in leads V1 to V4, normal p wave, normal PR interval, and normal QRS complex

His first ECG (Figure 1) showed a heart rate of 68 beats per minute, mild hyperacute T waves in V1 to V4, normal p waves, normal PR intervals, and a normal QRS complex. His 2D echogram revealed ischemic heart disease along with severe LV systolic dysfunction. A regional wall motion anomaly was found. The left ventricle's ejection fraction was 25- 30%. Mild to moderate Mitral Regurgitation (MR), trivial Aortic Regurgitation (AR), mild to moderate Tricuspid Regurgitation (TR), and mild Pulmonary Artery Hypertension (PAH) were also seen.

**Figure 2:**
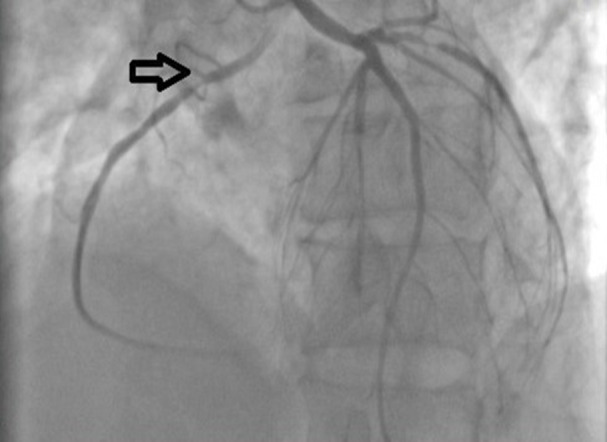
Figure 2. Coronary Angiography demonstrates an anomalous Right Coronary Artery originating from the Left Ostium and significant stenosis due to an atherosclerotic lesion in the proximal RCA

His symptoms improved once he began taking aspirin, clopidogrel, atorvastatin, heparin, and noradrenaline for hypotension. Coronary angiography revealed an anomalous Right Coronary Artery originating from the Left Ostium, with 90% stenosis in the proximal RCA (Figure 2). The left main coronary artery was normal, but the left anterior descending artery had a patent stent. The left circumflex had 90% proximal stenosis.

A blood hemoglobin level of 10.8 g/dL (normal range: 12 to 15.5 g/dL) was discovered during the laboratory assessment, indicating moderate anemia; creatinine levels were within normal ranges. His HIV and Hepatitis B test results were negative.

**Figure 3:**
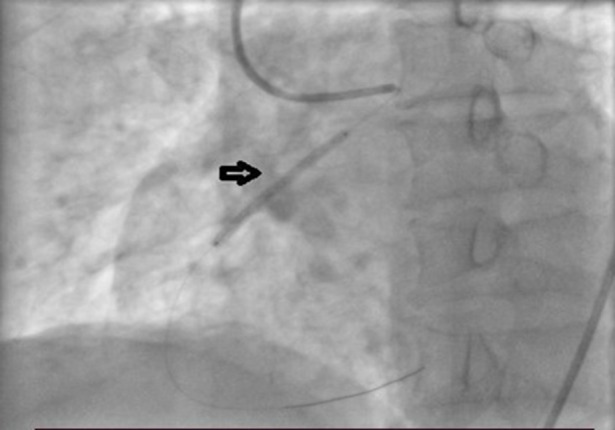
Figure 3. Wiring was done with a hydrophilic wire and Drug-eluting Stent deployment in an anomalous RCA

**Figure 4:**
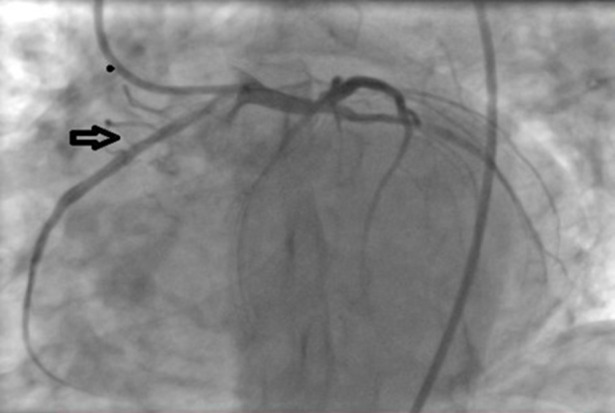
Figure 4. Successful deployment of the stent and TIMI 3 flow post-stenting

Interventional cardiologists performed angioplasty using a stent implant in the right coronary artery (Figure 3) and the left circumflex artery. A final angiography showed that the treatment was effective (Figure 4).

The patient was discharged on the third day.

## DISCUSSION

The patient had a severe medical history, including acute renal injury, a prior myocardial infarction, and cardiogenic shock. Nonetheless, the medical team's quick reaction, thorough examination, and successful coronary angioplasty with stent placement were agreed upon. Chronic risk factors, such as a history of tobacco use, the presence of severe left ventricular systolic dysfunction, and a delay in seeking medical attention, are limitations. The patient's anemia, as well as his lack of information about psychological health, exacerbates the condition. Though the medical regimen and treatments provided symptom alleviation, persisting risks and the likelihood of recurrence remain. This example emphasizes the need to recognize symptoms early on, address lifestyle factors, and create comprehensive, long-term management regimens that include regular follow-up after discharge and psychological support. The SCARE 2023 criteria reported the work^5^.

The anomalous beginning of the right coronary artery (RCA) is a rare congenital abnormality, with the first report dating back to 1948 by White and Edward^6^.

Physical examination and diagnostic tests are usually non-revealing in the absence of myocardial infarction or symptoms of chronic ischemia^7^. When a suspected anomalous coronary artery origin is discovered, echocardiography may help confirm the diagnosis^8^. To evaluate atypical coronary arteries, physicians use a range of diagnostic procedures, including cardiac catheterization, magnetic resonance angiography (MRA), and multidetector computed tomography (MDCT). Together, these procedures result in a comprehensive examination^9^.

With a three-dimensional reconstruction of the vessels, non-invasive modern imaging methods such as magnetic resonance imaging (MRI) and multidetector computed tomography (MDCT) allow us to explore the origin and path of the abnormal vessels. This information allows surgeons and interventional cardiologists to make an informed decision on the best course of therapy^10-12^ AAOCA can occur in either the right or left coronary artery and can take one of five frequent courses: intraarterial, retro aortic, subpulmonic (septal), pre-pulmonic, or retrocardiac. The interatrial course of the ACA—the segment that connects the aorta to the pulmonary artery—is the most clinically relevant because it is associated with angina and sudden mortality^13^.

Surgery, percutaneous intervention (stent installation), pharmacological therapy, and observation are among the therapeutic options^14^. Complications include arrhythmias, myocardial ischemia, and rapid cardiac arrest or death^15^. The detection of cases of aberrant aortic origin of the right coronary artery (AAORCA) is increasing as cardiac CT scans and coronary angiograms are more often used. Identifying mild symptoms is crucial for preventing significant morbidity.

## CONCLUSIONS

This case demonstrates the complex interplay between a rare congenital defect, severe cardiovascular diseases, and the influence of chronic risk factors on an individual's health. The 75-year-old male patient's history of tobacco use, along with a complicated medical history that included acute renal damage, previous myocardial infarction, and cardiogenic shock, presented complications. The example also highlights the limits and ongoing hazards associated with chronic conditions such as delayed medical treatment and severe left ventricular systolic failure. Despite symptomatic relief from a well-planned medication regimen, continuing hazards and the possibility of recurrence remain. The patient's mild anemia, along with a lack of information about her psychological state, complicates the clinical picture.

Moving forward, increased awareness of congenital defects, new diagnostic procedures, and a multidisciplinary approach will be critical to improving the prognosis and quality of life for those with complicated cardiovascular problems.

## Bullet Points

*· Rare congenital abnormalities such as AAORCA often present challenges in diagnosis and treatment*.

*· The successful management of the patient’s condition involved a multidisciplinary approach*.

*· The usage of accurate diagnostic methods, such as ECG, Echocardiogram and Angiography highlights the importance of accurately identifying and treating anomalous coronary artery origins*.

## References

[1] Kimbiris D, Iskandrian A S, Segal B L, Bemis C E (1978). Anomalous aortic origin of coronary arteries.. Circulation.

[2] Brothers Julie, Gaynor J William, Paridon Stephen, Lorber Richard, Jacobs Marshall (2009). Anomalous aortic origin of a coronary artery with an interarterial course: understanding current management strategies in children and young adults.. Pediatric cardiology.

[3] Neufeld HN, Schneeweiss A (1983). Coronary Artery Disease in Infants and Children. Lea Febiger, Philadelphia.

[4] Taylor A J, Rogan K M, Virmani R (1992). Sudden cardiac death associated with isolated congenital coronary artery anomalies.. Journal of the American College of Cardiology.

[5] Sohrabi Catrin, Mathew Ginimol, Maria Nicola, Kerwan Ahmed, Franchi Thomas, Agha Riaz A (2023). The SCARE 2023 guideline: updating consensus Surgical CAse REport (SCARE) guidelines.. International journal of surgery (London, England).

[6] WHITE N K, EDWARDS J E (1948). Anomalies of the coronary arteries; report of four cases.. Archives of pathology.

[7] Chaitman B R, Lespérance J, Saltiel J, Bourassa M G (1976). Clinical, angiographic, and hemodynamic findings in patients with anomalous origin of the coronary arteries.. Circulation.

[8] Frommelt Peter C, Frommelt Michele A, Tweddell James S, Jaquiss Robert D B (2003). Prospective echocardiographic diagnosis and surgical repair of anomalous origin of a coronary artery from the opposite sinus with an interarterial course.. Journal of the American College of Cardiology.

[9] Moza Ankush, Prashar Rohini, Bawany Muhammad (2011). Anomalous origin of right coronary artery associated with hypertrophic obstructive cardiomyopathy.. The American journal of the medical sciences.

[10] Lorber Richard, Srivastava Shubhika, Wilder Travis J, McIntyre Susan, DeCampli William M, Williams William G, Frommelt Peter C, Parness Ira A, Blackstone Eugene H, Jacobs Marshall L, Mertens Luc, Brothers Julie A, Herlong J René (2015). Anomalous Aortic Origin of Coronary Arteries in the Young: Echocardiographic Evaluation With Surgical Correlation.. JACC. Cardiovascular imaging.

[11] Warnes Carole A, Williams Roberta G, Bashore Thomas M, Child John S, Connolly Heidi M, Dearani Joseph A, Del Nido Pedro, Fasules James W, Graham Thomas P, Hijazi Ziyad M, Hunt Sharon A, King Mary Etta, Landzberg Michael J, Miner Pamela D, Radford Martha J, Walsh Edward P, Webb Gary D (2008). ACC/AHA 2008 guidelines for the management of adults with congenital heart disease: a report of the American College of Cardiology/American Heart Association Task Force on Practice Guidelines (Writing Committee to Develop Guidelines on the Management of Adults With Congenital Heart Disease). Developed in Collaboration With the American Society of Echocardiography, Heart Rhythm Society, International Society for Adult Congenital Heart Disease, Society for Cardiovascular Angiography and Interventions, and Society of Thoracic Surgeons.. Journal of the American College of Cardiology.

[12] Ripley David P, Saha Ansuman, Teis Albert, Uddin Akhlaque, Bijsterveld Petra, Kidambi Ananth, McDiarmid Adam K, Sivananthan Mohan, Plein Sven, Pennell Dudley J, Greenwood John P (2014). The distribution and prognosis of anomalous coronary arteries identified by cardiovascular magnetic resonance: 15 year experience from two tertiary centres.. Journal of cardiovascular magnetic resonance : official journal of the Society for Cardiovascular Magnetic Resonance.

[13] Lee Hye-Jeong, Hong Yoo Jin, Kim Hee Yeong, Lee Jiwon, Hur Jin, Choi Byoung Wook, Chang Hyuk-Jae, Nam Ji Eun, Choe Kyu Ok, Kim Young Jin (2012). Anomalous origin of the right coronary artery from the left coronary sinus with an interarterial course: subtypes and clinical importance.. Radiology.

[14] Lilly Steven M, Schussler Jeffrey M, Stoler Robert C (2011). Anomalous origin of the right coronary artery from the left sinus of Valsalva associated with syncope in a young athlete.. Proceedings (Baylor University. Medical Center).

[15] Pradhan Snehasis, Gresa Kciku, Trappe Hans-Joachim (2020). Anomalous right coronary artery with interarterial course depicting an unusual case of an electrical storm: a case presentation.. BMC cardiovascular disorders.

